# Impact of Tablet Shape on Drug Dissolution Rate Through Immediate Released Tablets

**DOI:** 10.34172/apb.2020.079

**Published:** 2020-08-09

**Authors:** Fatima Molavi, Hamed Hamishehkar, Ali Nokhodchi

**Affiliations:** ^1^Biotechnology Research Center, Student Research Committee and Faculty of Pharmacy, Tabriz University of Medical Sciences, Tabriz, Iran.; ^2^Drug Applied Research Center, Tabriz University of Medical Sciences, Tabriz, Iran.; ^3^Pharmaceutics Research Laboratory, School of Life Sciences, University of Sussex, Falmer, Brighton BN1 9QJ, United Kingdom.

**Keywords:** Dissolution modeling, Tablet, Drug release, Domperidone, Geometric properties

## Abstract

***Purpose:*** The aim of this study was to evaluate the influence of the geometric shape on the dissolution rate of the domperidone, a drug model for immediate release dosage form. In this regard, a lack of sufficient information about the effective dissolution rate of the drugs regarding their shapes has made this issue an interesting subject for researchers.

***Methods:*** For this purpose, three tablet shapes, namely flat and biconvex both in a round and oblong shapes, with different four sizes were modelled for the preparation of domperidone tablet. *In vitro* dissolution test was accomplished using a USP dissolution apparatus II. The drug dissolution rate was assessed by calculating various dissolution parameters; e.g., dissolution efficiency (DE), mean dissolution rate (MDR), mean dissolution time (MDT), and difference and similarity factors (f_1_ and f_2_ ).

***Results:*** Regarding the disintegration time, the larger tablets showed a faster disintegration time. When the size of the tablets was smaller, the amount of released drug was significantly decreased. In addition, #9 tablets with a flat or biconvex geometry had obvious effects on the DE values. Generally, biconvex tablets had higher DE percentage than the flat tablets.

***Conclusion:*** Noticeable differences in dissolution parameters by considering the different geometric shapes play an important role in the drug release kinetics which makes a significant effect on quick onset of action in oral administration.

## Introduction

Dissolution rate studies play a key role in the development of pharmaceutical dosage forms, *in vitro* and *in vivo* correlation (IVIVC) assessment,^1^ registration, and quality control of various dosage forms.^2^ The dissolution methods for individual drugs are determined by the solubility of the active substance,^3^ the dosage form characteristics,^4^ and the intended route of administration such as oral solid dosage form. Improving the dissolution rates of drugs by different techniques is an increasing demand of pharmaceutical industries and can be achieved by various methods including modifying physicochemical properties by preparation of nano-sized drug particles,^5,6^ solid dispersions approach,^7^ particle design^8^ and adsorption onto pharmaceutical diluents.^9^ It has been proved that the *in vitro* dissolution rate is proportional to *in vivo* absorption rate data.^1,10^ Therefore, the prediction of drug dissolution is extremely important for formulators in pharmaceutical industries. Attracting appearance for marketing aspects and production of various tablet shapes for pediatric compliance have led to the creation of various tablet shapes.^11^ In addition, tablet shape is an important subject for pharmaceutical industrials because of its influence in product development, process operating conditions^12-14^ and marketing issues. Domperidone, a drug model for immediate release dosage form was selected according to its biopharmaceutical classification system that classified to class II, suggesting that the release of these type of drugs from dosage forms is easily controlled by formulation composition.^15^ Therefore, we can easily study and evaluate dissolution manner related to various tablet shapes. The objective of the present study was to investigate the impact of different tablet shapes on dissolution rate. By considering that the formulation composition and hardness, two main affecting factors on dissolution rate are the same in the tablets with various shapes. In addition as the novelty of the study, the results of this project will be able to guide pharmaceutical industry formulators to simulate the drug release pattern of generic formulations or adjust it according to the pharmacopoeia requirements.

## Materials and Methods

### Materials

Domperidone maleate (M/S Vasudha Pharma Chem Limited, India) was kindly donated from Zahravi Pharmaceutical Co. (Tabriz, Iran). Lactose monohydrate, Avicel^®^ PH-101, pregelatinized starch, and magnesium stearate were provided from DMV (Germany), Mingtai (Taiwan), Colorcon (USA), respectively. Hydrochloric acid and polysorbate 20 were purchased from Merck Chemicals Co. (Germany). Colloidal silicon dioxide was provided from Kirsch pharma (Germany).

### Methods

#### Formulation and tablet Preparation

**Figure 1:**
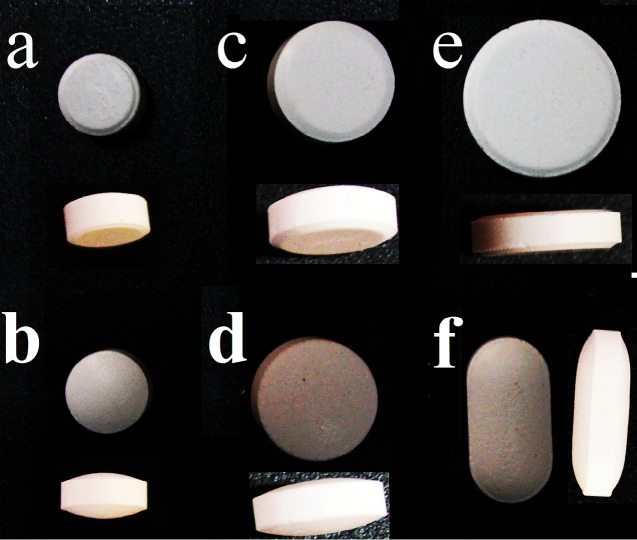
Domperidone tablets with different shapes: a) Round #6 flat, b) Round #6 biconvex, c) Round #9 flat, d) Round #9 biconvex, e) Round #11 flat, and f) Oblong #12 biconvex.

To prepare domperidone maleate tablets wet granulation method was employed. The required quantities of the ingredients were weighed and blended using the tumbling method to provide a homogenous granule mixture as summarized in Table 1. The granules were compressed on different punch and die shapes i.e. round #6 biconvex, round #6 flat, round #9 biconvex, round #9 flat, oblong #12 flat, round #11 flat (Figure 1). Tablets were pressed by a rotary compression machine (ERWEKA AR 400, Germany). Fifty tablets in each shape were manufactured containing different weights but the same powder composition with approximately equal hardness about 8-9 kPa.

**Table 1:** Formulation of domperidone maleate

**Composition**	**Percentage (%)**	**Function in the formulation**
Domperidone maleate	12.50	Active ingredient
Lactose monohydrate	51.83	Diluent
Avicel PH-101	24.55	Diluent and disintegrant
Pregelatinized starch	6.88	Diluent
Polysorbate 20	0.96	Solubilizing agent
Colloidal silicon dioxide	0.29	Glidant
Magnesium stearate	2.95	Lubricant
Total quantity	100.0	-

### Tablet characterization

Thickness, diameter and hardness were characterized using a hardness tester (Model, ERWEKA, Germany). Assay and content uniformity of tablets also were assessed according to British Pharmacopoeia (BP 2015).

Values of the total surface area of tablets either flat or biconvex are calculated by addition of surface on both sides of the tablet (SA) of radius (πr^2^) and spherical cap surface areas (2πRh). Where h is the height, R is the spherical cap radius and r is the base radius.

### Dissolution study

Dissolution test for domperidone tablets in the BP, briefly is described in Table 2. The area under the dissolution curve up to the time, t, is defined as the dissolution efficiency (DE).

$$DE\%=\frac{\int_{0}^{t}ydt}{y_{100}(t_{2}-t_{1})}\times 100$$

**Table 2:** Dissolution test conditions for domperidone tablets according to BP (2015)

**Apparatus**	**Paddle**
Medium	HCl 0.1 N
Speed	50 rpm
Procedure	UV (λ=286nm)
Time	2, 5, 8, 10, 15, 20, 30, and 45 minutes

Where y is the percentage of domperidone dissolved at time t.^16^

The mean dissolution time (MDT) is another parameter to explain the drug dissolution rate from a solid state of a dosage form.

$$MDT=\frac{\sum_{i=1}^{n}t_{i}\Delta M_{i}}{\sum_{i=1}^{n}\Delta M_{i}}$$

Where i is the total number of dissolution sample times, ΔM_i_ is the added amount of drug dissolved between t_i_ and t-1; and t_i_ is the midpoint time between two samples in the t and t-1.

Another parameter that represents the dissolution rate is the mean dissolution rate (MDR) as an independent metric. It can be calculated according to the following equation, in addition showing the mean percentage of drug dissolved to time.

$$MDR=\frac{\sum_{i=1}^{n}\frac{\Delta M_{i}}{\Delta t}}{n}$$

Where n is the number of sample time points, Δt is the midpoint time.^17^

The similarity factor f_2_ and the difference factor f_1_ were calculated according to the following equations:

$$\begin{array}{l} f_{2}=50.\log\left[\frac{100}{\sqrt{1+\frac{\sum_{t=1}^{t=n}{\left[R_{t}-T_{t}\right]}^{2}}{n}}}\right]\\ f_{1}=[\sum_{t=1}^{n}\left(R_{t}-T_{t}\right)[\sum_{t=1}^{n}R_{t}]\times 100 \end{array}$$

Where R_t_ and T_t_ are the refrence profile and the test profile of cumulative percentage of drug dissolved at time point t, respectively. To consider dissolution profiles as similar and bioequivalent, the value of f_2_ and f_1_ should be between 50 through 100 and lower than 10, respectively.^18^

To study drug-release kinetics, several dissolution models such as zero order, first order, Higuchi, Hixson–Crowell, Weibull, and Korsmeyer and Peppas has well-known.^19,20^ The accurate fitting model was selected based on R-squared (RSQ) and mean percent error (E) from drug release data.^21^

### Statistical analysis

Independent comparison models and ANOVA based on statistical methods has been performed to compare dissolution profiles. In this procedure, Tukey test was applied as independent analysis model, dominating the multiple comparison tests.

## Results and Discussion

Domperidone tablets were prepared through wet granulation technique using scale-up equipment to reach an acceptable appearance and same hardness. Table 3 shows the characteristics of the appearance of tablets with the same formulation. The results have shown that the hardness of tablets is almost similar. Tablet shapes and dissolution rate are considered as independent and dependent variables, respectively. The results of the assay and content uniformity of tablets have been reported as acceptable. The result of active pharmaceutical ingredient assay was 98-102% and the acceptance value (AV) for content uniformity test was around 4-5, where AV≤15 is considered as suitable.

**Table 3:** Description for different tablets

**Appearance Shape**	**Weight (mg)**	**Thickness (mm)**	**Diameter (mm)**	**Area (mm** ^ 2 ^ **)**	**Hardness (kp** ^a^ **)**
Round #6 biconvex	102 ± 0.6	3.08 ± 0.01	6.04 ± 0.02	113.84	9.1 ± 0.1
Round #6 flat	102 ± 0.1	2.70 ± 0.01	6.06 ± 0.01	95.56	8.8 ± 0.3
Round #9 flat	250 ± 0.7	3.09 ± 0.01	9.02 ± 0.01	214.88	9.9 ± 0.4
Round #9 biconvex	251 ± 1.4	3.70 ± 0.05	9.06 ± 0.06	231.84	8.5 ± 0.8
Round #11 flat	394 ± 2.2	3.05 ± 0.02	11.06 ± 0.02	321.59	8.7 ± 0.6
Oblong #12 biconvex	253.8 ± 0.7	3.83 ± 0.01	12.16 × 6.15 ± 0.0	>400	6.9± 0.5

Data was presented as mean ± standard deviation (n=6). ^a^kp, kilopond.

### Model-independent approaches

The aim of this study was to characterize the different geometric types of tablets on domperidone release. To evaluate dissolution behavior and to describe the relationship between drug release behaviors, the main dissolution parameters i.e., DE%, MDT, MDR, and disintegration time were studied. By comparing the results, the dissolution test parameters are completely changed by changing the appearance of the tablets. The results, as shown in Table 4, indicated that larger tablets had faster disintegration time. The oblong #12 biconvex tablets had the fastest disintegration time, which would be desired in its pharmacological performance. In addition, biconvex tablets demonstrated a faster drug release than flat tablets. This fact is more significant in larger tablets probably because of providing more surface area for tablets. The results of other time-dependent parameters (MDT and MDR) were well matched with the results of disintegration time. The high disintegration time and low DE% in round #6 tablets matrix can be attributed to its low surface area. As the formulation of tablets is the same, according to the Noyes and Whitney equation^22^ a low surface area between the solute and the solvent leads to a reduction in the amount of dissolved substance. In fact, the smaller size of tablets leads to significantly decreased percent of DE. In addition, flat and biconvex (#9) tablets showed high DE values and comparing between flat and biconvex tablets, biconvex tablets exhibited a higher DE value. Statistical analysis was carried out using one-way ANOVA based on DE data, this result was insignificant at the p = 0.319 level for #6 flat and biconvex tablets. It revealed that the release profiles of these shapes are truly similar. Besides, p-value for #6 flat and #9 flat tablets is less than 0.003, suggesting that the size of tablets is a very important parameter in dissolution behavior. In addition, by comparing drug dissolution between #9 flat-faced and biconvex tablets, at the *P* = 0.0001 level, signifying that by increasing in the size of tablets, the difference between flat-faced tablets and biconvex tablets becomes more significant. Furthermore, different types of tablets have shown different initial release which is important in immediate-release tablets to start the biological effect as early as the drug was taken.

**Table 4:** Dissolution parameters

**Sample**	**Disintegration time (min)**	**DE (%)**	**MDT**	**MDR**
Round #6 biconvex	15:20”± 01:30”	73.71± 1.94	18.85 ± 1.40	1.36 ± 0.07
Round #6 flat	14:05”± 01:00”	73.80± 1.89	18.78 ± 1.37	1.11 ± 0.08
Round #9 flat	7:19” ± 01:30”	88.59± 1.49	8.44 ± 1.04	2.36 ± 0.20
Round #9 biconvex	3:52” ± 01:30”’	91.95± 2.14	6.10 ± 1.47	3.49 ± 0.91
Round #11 flat	6:03” ± 01:00”	88.75± 1.94	8.36 ± 1.38	2.38 ± 0.34
Oblong #12 biconvex	1:40” ± 00:30”	90.54± 1.81	7.14 ± 1.30	4.07 ± 0.21

DE, Dissolution efficiency; MDR, Mean dissolution rate; MDT, Mean dissolution time. Data was presented as mean ± standard deviation (n=6).

**Figure 2:**
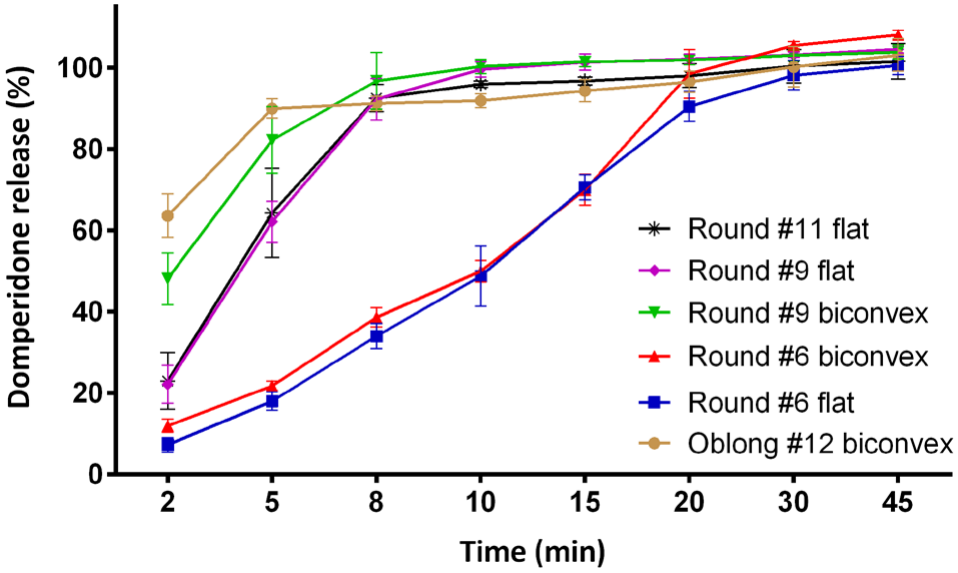
Dissolution profiles of different shapes of tablets. Values were presented in mean with standard deviation (n=6).

As the size of the tablets increased, the burst release was faster. It reveals that in addition to the effect of hardness of the tablet on the disintegration and the dissolution rate of the tablet, the shape of the tablet also has a significant effect which makes the quick onset of action in oral administration. A similar study performed on metronidazole sustained dosage forms also presented that surface area can be used as a factor to estimate the drug release profile.^23^ A study on the effect of different geometrical shapes i.e. triangular, cylindrical and half-spherical on theophylline release indicated that the highest releases were observed for triangular erodible tablets in 1:1 drug/polymer ratio and for half-spherical tablets with 1:0.5 drug/polymer ratio.^24^ The results of this study proved that the geometric shape has an effect on the diffusion and release kinetics. The results obtained from the analysis of drug release of the different geometric shapes of tablets are shown in Figure 2. It reveals that there has been a different dissolution profile among the products. Burst release plays the main role in the successful therapeutic performance of anti-vomiting dosage forms. Tablets in various shapes provide different burst releases. The order of domperidone burst release from various tablets were Round #6 flat < Round #6 biconvex <Round #9 flat < Round #11 flat < Round #9 biconvex < Oblong #12 biconvex. It is concluded that a decrease in the size of tablets significantly reduces the drug burst release. Interestingly, biconvexity improved the burst release and helped fulfilment of complete drug release from tablet dosage forms. Statistical approaches, f_1_ (the difference factor) and f_2_ (the similarity factor) were used to compare the dissolution profiles. Table 5 presents the experimental data for the comparison of the individual dissolution profiles. As can be seen, tablet size has a key role in the drug release behavior of tablets. The varying surface area can change the dissolution rate of non-flat geometry of solid dosage forms.^25^ Although comparing round #6 biconvex and round #6 flat tablets demonstrated that being flat and biconvex in small tablets is not important for drug dissolution, but this comparison for #9 tablets indicated the opposite results. This observation confirmed that the drug release behavior are affected by being flat and biconvex in larger tablets.

**Table 5:** Domperidone release profile comparison between different shape tablets through difference (f_1_) and similarity (F_2_) factors

**Tablet shape**	**f**_1_ **(%)**	**f**_2_ **(%)**
Round #6 biconvex vs. Round #6 flat	7.49	62.97
Round #9 flat vs. Round #9 biconvex	7.62	46.47
Round #9 flat vs. Round #6 flat	31.93	23.40

### Model-dependent approaches

Mathematical models based on statistical analysis was used to characterize the dissolution profiles.^26^ Model dependent approaches due to the complexity of*in vitro* methods have received considerable attention. The minimum errors and RSQ around 100 between the fitted and the actual data are acceptance conditions to apply dissolution models. Here, only Korsmeyer-Peppas model describes accurately the release data of round #6 flat, round #6 biconvex, and round #9 flat domperidone tablets with RSQ of 0.97, 0.99, and 1 with an error of 26.34, 30.00, and 0.00%, respectively. Other shapes cannot be defined by any of the models as the large burst release (60%) happened in the first minutes. Korsmeyer-Peppas model is expected to be successfully applied to the analysis of drug release kinetics from homogenous and dissolving tablet matrix in 60% of the initial release. To the best of our knowledge, these models were justified theoretically via unification of the Fick’s first law of diffusion and the Noyes- Whitney law of dissolution. As result, according to Korsmeyer-Peppas model, the release mechanism for small flat tablets, #6 and relatively #9, in addition to diffusion, erosion of tablets can also be considered in the first minutes; as these results match those observed in the disintegration study.

## Conclusion

Since the emerge of the Noyes-Whitney equation in 1897, dissolution research has been initiated and still now it is a critical issue in the chemistry.^27^ In this regard, the dissolution test is an essential component of drug development in the pharmaceutical industry. This research evaluated the effect of six different tablet shapes on the dissolution rate. The current study indicated that by decreasing the size of tablets, the drug burst release was significantly decreased. Although the present study is based on a small sample of tablet shapes, the findings indicate that the initial burst and complete release occurred in large, biconvex tablets. Further research regarding the role of tablet shapes would be worthwhile.

## Ethical Issues

Not applicable.

## Acknowledgments

The research reported in this publication was supported by Elite Researcher Grant Committee under award number 958777 from the National Institutes for Medical Research Development (NIMAD), Tehran, Iran.

## Conflict of Interest

The authors confirm that this article has no conflict of interest.
